# Expression of the Components of the Renin–Angiotensin System in Venous Malformation

**DOI:** 10.3389/fsurg.2016.00024

**Published:** 2016-05-03

**Authors:** Sam Siljee, Emily Keane, Reginald Marsh, Helen D. Brasch, Swee T. Tan, Tinte Itinteang

**Affiliations:** ^1^Gillies McIndoe Research Institute, Wellington, New Zealand; ^2^University of Auckland, Auckland, New Zealand; ^3^Department of Pathology, Hutt Hospital, Wellington, New Zealand; ^4^Centre for the Study and Treatment of Vascular Birthmarks, Wellington Regional Plastic, Maxillofacial and Burns Unit, Hutt Hospital, Wellington, New Zealand

**Keywords:** venous, malformation, renin, angiotensin, system, pro-renin, receptors

## Abstract

**Background:**

Venous malformation (VM) is the most common form of vascular malformation, consisting of a network of thin-walled ectatic venous channels with deficient or absent media. This study investigated the expression of the components of the renin–angiotensin system (RAS), namely, (pro)renin receptor (PRR), angiotensin-converting enzyme (ACE), angiotensin II receptor 1 (ATIIR1), and angiotensin II receptor 2 (AIITR2) in subcutaneous (SC) and intramuscular (IM) VM.

**Materials and methods:**

SC (*n* = 7) and IM (*n* = 7) VM were analyzed for the expression of PRR, ACE, ATIIR1, and ATIIR2 using 3,3-diaminobenzidine and immunofluorescent (IF) immunohistochemical (IHC) staining and NanoString gene expression analysis.

**Results:**

IHC staining showed expression of PRR, ACE, and ATIIR1, and faint expression of ATIIR2 in the endothelium of SC and IM VM. Furthermore, ATIIR2 was expressed by cells away from the endothelium in both SC and IM VM lesions examined. NanoString analysis demonstrated the presence of PRR, ACE, and ATIIR1 but not ATIIR2.

**Conclusion:**

The presence of PRR, ACE, ATIIR1, and potentially ATIIR2, in both SC and IM VM, suggests a role for the RAS in the biology of VM. This novel finding may lead to a mechanism-based therapy for VM.

## Introduction

Vascular anomalies are classified by the International Society for the Study of Vascular Anomalies classification system into vascular tumors and vascular malformations (1). Infantile hemangioma (IH) is the most common type of vascular tumor (1). Vascular malformation affects arteries, veins, lymphatics, and capillaries singly or in combinations, with venous malformation (VM) being the most common (1).

Venous malformation affects 1.5% of the population (2) and is characterized by thin-walled ectatic venous channels lined by flat endothelial cells (EC) with deficient or absent smooth muscle cells (SMC) (3, 4). VM may involve any body site and tissue (4), commonly in subcutaneous (SC) and less commonly in intramuscular (IM) locations (5). Although VM is present at birth, it may not become apparent until later in life (3–5). Its clinical presentation depends on the location and size of the lesion. SC lesions typically present as compressible masses with a bluish hue, whereas IM lesions often present with a swelling and/or pain (4). VM may cause cosmetic concerns and/or functional deficits, such as obstructive sleep apnea as in the case of oropharyngeal lesions (6). VM grows proportionately with the growth of the child and may suddenly expand in response to hormonal changes or trauma, including incomplete surgical excision (3–5).

About 1–2% of VM cases are familial caused by a TIE2 mutation (7). Half of the sporadic cases also consists a TIE2 somatic mutation (7). These mutations have been shown to result in ligand-independent hyper-phosphorylation of the TIE2 receptor (7). Vikkula et al. (8) suggest that the TIE2 mutation in EC in VM may reduce SMC ligand expression causing a local uncoupling between normal SMC recruitment and the proliferation of EC.

Furthermore, more recent demonstration of hormone receptor proteins, such as follicle-stimulating hormone receptor (9) and the growth hormone receptor (10) within VM, highlights other potential pathways that may play a critical role in the biology of VM.

The classical renin–angiotensin system (RAS) consists of the enzyme renin, which converts angiotensinogen to angiotensin I (ATI) (11). ATI is subsequently cleaved by angiotensin-converting enzyme (ACE), also known as CD143, to form angiotensin II (ATII) (11). The vasoactive peptide ATII then acts on angiotensin II receptor 1 (ATIIR1) and angiotensin II receptor 2 (ATIIR2) (11).

Previous work has identified the presence of primitive mesodermal cells with a neural crest phenotype in IH (12), regulated by the RAS (13). These discoveries of the stem cell nature of IH, regulated by the RAS, underscore the natural involution and accelerated involution of this tumor, induced by β-blockers and ACE inhibitors (14–16).

This study aimed to identify the expression of the components of the RAS, namely, PRR, ACE, ATIIR1, and ATIIR2, in both SC and IM VM.

## Materials and Methods

### Tissues

Subcutaneous VM tissues from seven patients aged 9–30 years (mean, 22.2 years) and IM VM from seven patients aged 16 months–54 years (mean, 21.3 years) were sourced from the Gillies McIndoe Research Institute Tissue Bank, in accordance with a protocol approved by the Central Health and Disability Ethics Committee (ref. no. 13/CEN/130). The tissues used in this study were confirmed to be SC or IM VM by a consultant anatomical pathologist (HDB) using hematoxylin and eosin (H&E) staining and D2-40 staining to exclude lymphatic malformation.

### Immunohistochemical Staining

3,3-Diaminobenzidine (DAB) and immunofluorescent (IF) immunohistochemical (IHC) staining were performed on 4-μm-thick formalin-fixed paraffin-embedded sections of VM samples as reported previously (17). In brief, IHC staining was undertaken using the Leica Bond Rx auto-stainer (Leica, Nussloch, Germany) with antibodies against CD34 (ready-to-use; cat#PA0212, Leica), ERG (1:200; cat#EP111, Cell Marque, Santa Cruz, CA, USA), SMA (ready-to-use; cat#PA0943, Leica), D2-40 (1:100; cat#M3619, Dako, Glostrup Denmark), PRR (1:100; cat#HPA003156, Sigma), ACE (1:40; cat#3C5, Serotec, Raleigh, NC, USA), ATIIR1 (1:25; cat#ab9391, Abcam, Cambridge, MA, USA), and ATIIR2 (1:2000; cat#NBPI-77368, Novus Biologicals, Littleton, CO, USA). All antibodies were diluted in Bond primary diluent (Leica). For IF IHC detection, a combination of Vectafluor Excel anti-rabbit 594 (ready-to-use; cat#VEDK-1594, Vector Laboratories, Burlingame, CA, USA) and Alexa Fluor anti-mouse 488 (1:500; cat#A21202, Life Technologies, San Diego, CA, USA) were used to detect combinations that included PRR and ATIIR2, and Vectafluor Excel anti-mouse (ready-to-use; cat#VEDK2488, Vector Laboratories) and Alexa Fluor anti-rabbit 594 (1:500; cat#A21207, Life Technologies) to detect combinations that included ACE and ATIIR1.

All DAB IHC-stained slides were mounted in Surgipath Micromount (cat#3801732, Leica). All IF IHC-stained slides were mounted in Vectashield HardSet antifade mounting medium with DAPI (cat#H-1500, Vector Laboratories).

Positive control samples were selected based on the previously reported expression of the relevant proteins: placenta for PRR (18), kidney for ACE (19) and ATIIR2 (20), and liver for ATIIR1 (20). To determine the specificity of the primary antibodies, staining of VM sections was performed by omitting the primary antibodies.

### Image Analysis

All DAB and IF IHC-stained slides were viewed and imaged using the Olympus BX53 microscope fitted with an Olympus DP21 digital camera (Olympus, Tokyo, Japan) and an Olympus FV1200 biological confocal laser-scanning microscope (Olympus, Tokyo, Japan), respectively. IF IHC images were processed with cellSens Dimension 1.11 software using 2D deconvolution algorithm (Olympus).

### NanoString Gene Expression Analysis

Total RNA was extracted from ~10 mg of snap-frozen IM (*n* = 3) and SC (*n* = 3) VM tissues from the same cohorts of patients included in DAB IHC staining, using RNeasy Mini Kit (Qiagen, Hilden, Germany) and quantitated with the NanoDrop 2000 Spectrophotometer (Thermo Scientific). The samples with A260/A230 ≥1.8 and A260/A280 ≥1.9 were used for further analysis. The integrity of the RNA was assessed by Agilent 2100 BioAnalyser (Agilent Technologies, Santa Clara, CA, USA). The isolated RNA was then subjected to NanoString nCounter™ Gene Expression Assay (NanoString Technologies, Seattle, WA, USA) and completed by New Zealand Genomics, Ltd. (Dunedin, New Zealand), according to the manufacturer’s protocol. Probes for the genes encoding PRR (ATP6AP2, NM_005765.2), ACE (NM_000789.2), ATIIR1 (NM_000685.3), ATIIR2 (NM_000686.3), and the housekeeping gene, GAPDH (NM_002046.3) were designed and synthesized by NanoString Technologies.

### Statistical Analyses

Raw NanoString data were analyzed using SPSS (v22, IBM), corroborated with nSolver™ software (NanoString Technologies) using standard settings, and normalized against the housekeeping gene. To determine the level of confidence between the IM and SC VM samples, two-tailed Student’s *t*-test was performed. Charts were made with Excel (Microsoft Office 2013).

## Results

### Histochemical and DAB Immunohistochemical Staining

**Figure 1:**
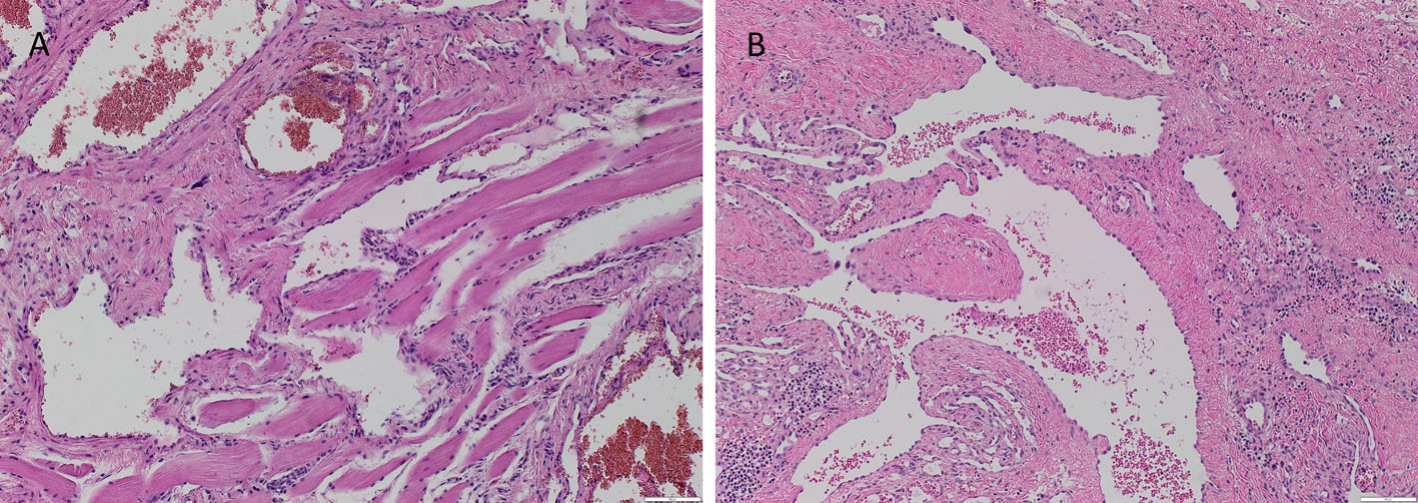
**Representative H&E stained slides of IM (A) and SC (B) VM showing thin-walled ectatic lesional vessels with variable density of smooth muscle cells**. Original magnification: 100×.

The vasculature within the VM lesions was identified by H&E staining, characterized by thin-walled ectatic venous channels with deficient or absent SMC in both IM (Figure 1A) and SC (Figure 1B) VM. None of the IM or SC VM lesions used in this study expressed D2-40 (Figures S1A,B in Supplementary Material, brown).

**Figure 2:**
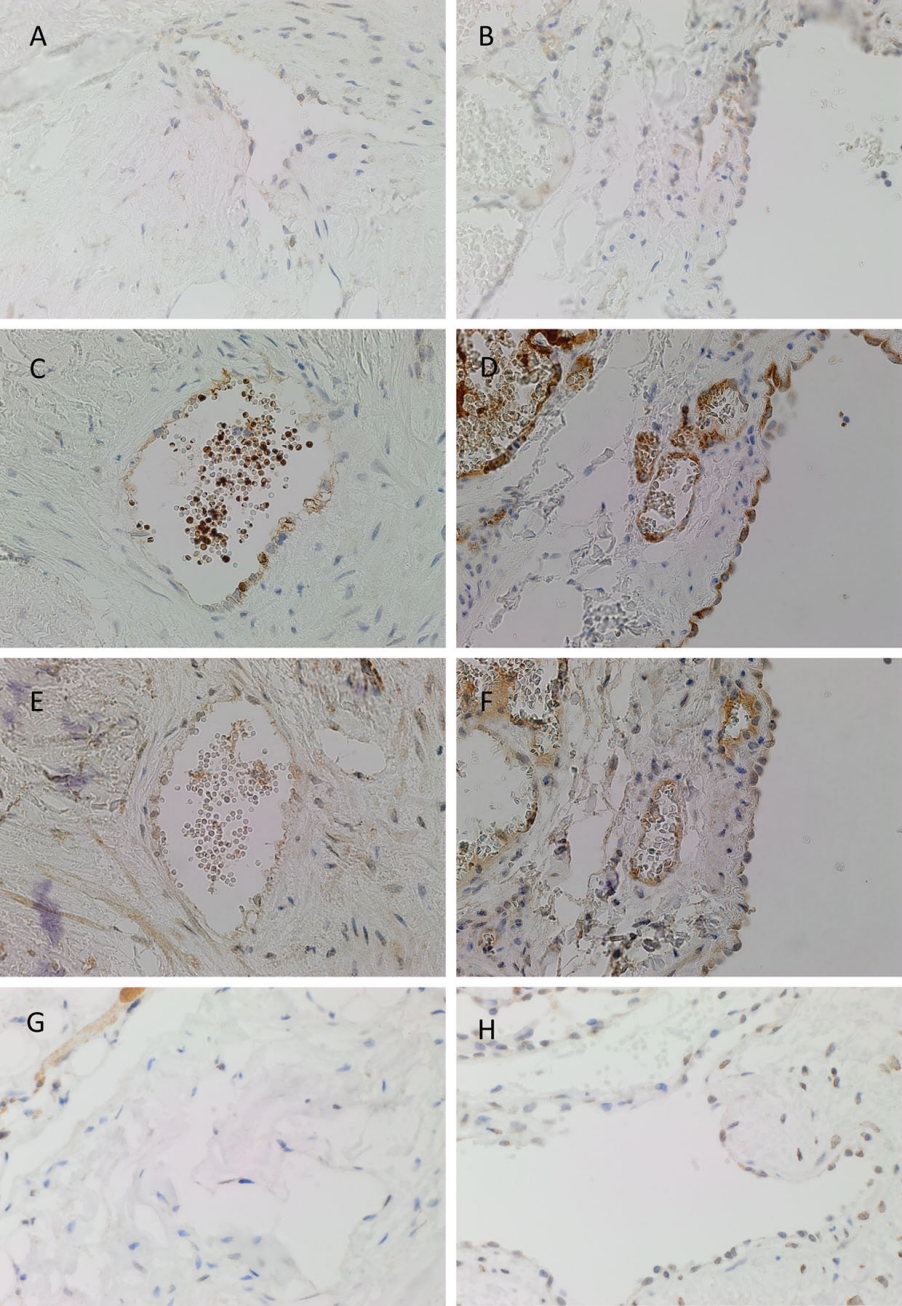
**Representative DAB IHC-stained sections of IM [(A,C,E,G), brown] and SC [(B,D,F,G), brown] VM demonstrating the expression of PRR in IM [(A), brown] and SC [(B), brown] lesions**. ACE was also expressed on the endothelium of IM [**(C)**, brown] and SC [**(D)**, brown] lesions. ATIIR1 was expressed in both IM [**(E)**, brown] and SC [**(F)**, brown] lesions. Faint staining of ATIIR2 was seen on the endothelium of IM [**(G)**, brown] and SC [**(H)**, brown] VM. Original magnification: 400×.

PRR was expressed on the endothelium of both IM (Figure 2A, brown) and SC (Figure 2B, brown) VM. ACE, which converts ATI to ATII, was expressed on the endothelium of IM (Figure 2C, brown) and SC (Figure 2D, brown) VM. To demonstrate a potential receptor available for downstream signaling of ATII, staining for ATIIR1 and ATIIR2 was performed. This demonstrated the expression of ATIIR1 on the endothelium of IM (Figure 2E, brown) and SC (Figure 2F, brown) VM. There was faint staining of ATIIR2 of the endothelium and cells away from the endothelium of both IM (Figure 2G, brown) and SC (Figure 2H, brown) VM lesions studied.

**Figure 3:**
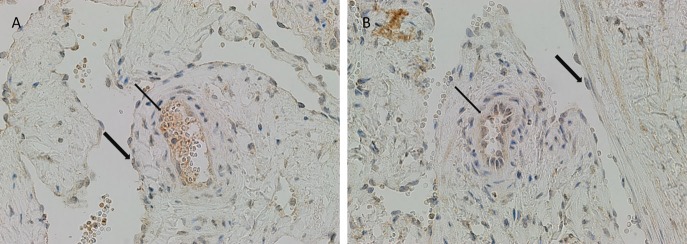
**ATIIR1 was expressed to a greater degree in the smaller lesional vessels [(A,B), brown, *thin arrow*], compared with the adjacent dilated lesional vessels [(A,B), brown, *thick arrow*] in both IM (A) and SC (B) VM**. Original magnification: 400×.

There was relatively more intense staining for ATIIR1, in both IM and SC VM, in the smaller lesional vessels (Figures 3A,B, brown, *thin arrow*) compared to the dilated vessels (Figures 3A,B, brown, *thick arrow*).

Positive controls for PRR (Figure S2A in Supplementary Material, brown), ACE (Figure S2B in Supplementary Material, brown), ATIIR1 (Figure S2C in Supplementary Material, brown), ATIIR2 (Figure S2D in Supplementary Material, brown), and negative control with omission of the primary antibodies (Figure S2E in Supplementary Material, brown) demonstrated appropriate specificity for the antibodies used.

### Immunoflourescent Immunohistochemical Staining

**Figure 4:**
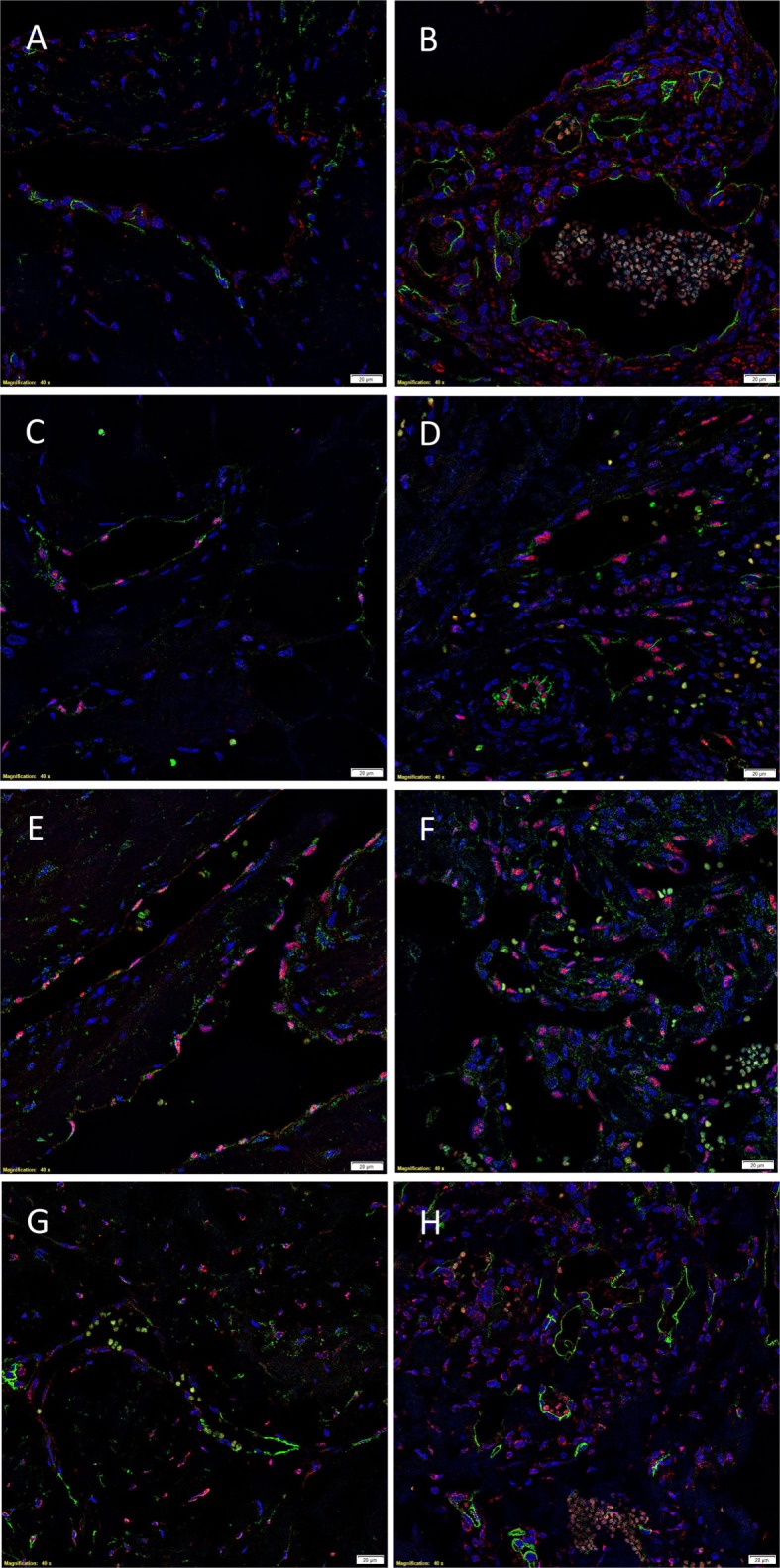
**Representative IF IHC-stained sections of IM (A,C,E,G) and SC (B,D,F,H) VM demonstrating the expression of PRR in IM [(A), red] and SC [(B), red] lesions**. Endothelial cells were identified by the endothelial marker CD34 [**(A,B)**, green]. ACE was also expressed on the endothelium of IM [**(C)**, green] and SC [**(D)**, green] lesions. ATIIR1 was expressed on the endothelium of both IM [**(E)**, green] and SC [**(F)**, green] VM. The endothelial cells were identified using the endothelial marker ERG [**(C–F)**, red]. ATIIR2 also demonstrated positive staining in IM [**(G)**, red] and SC [**(H)**, red] VM. The endothelial cells were identified using CD34 [**(G,H)**, green]. Cell nuclei were counterstained with 4′,6-diamidino-2-phenylindole (blue). Scale bars: 20 μm.

The endothelium of all IM (Figures 4A,C,E,G) and SC (Figures 4B,D,F,H) VM were identified by either CD34 (Figures 4A,B,G,H, green) or ERG (Figures 4C–F, red). IF IHC staining demonstrated the expression of PRR on the endothelium of IM (Figure 4A, red) and SC (Figure 4B, red) VM. The expression of ACE was demonstrated on the endothelium of IM (Figure 4C, green) and SC (Figure 4D, green) VM. ATIIR1 was expressed on the endothelium of IM (Figure 4E, green) and SC (Figure 4F, green) VM. ATIIR2 was also demonstrated on cells of the endothelium as well as cells away from the endothelium in both IM (Figure 4G, red) and SC (Figure 4H, red) VM. Cell nuclei were highlighted in blue (Figures 4A–H). Individual IF IHC staining for each of the aforementioned proteins is shown in Figure S3 in Supplementary Material.

Negative controls for IF IHC staining demonstrated appropriate specificity of the primary antibodies in anti-mouse (Figure S4A in Supplementary Material, green) and anti-rabbit (Figure S4B in Supplementary Material, red) combinations.

### NanoString Gene Analysis

**Figure 5:**
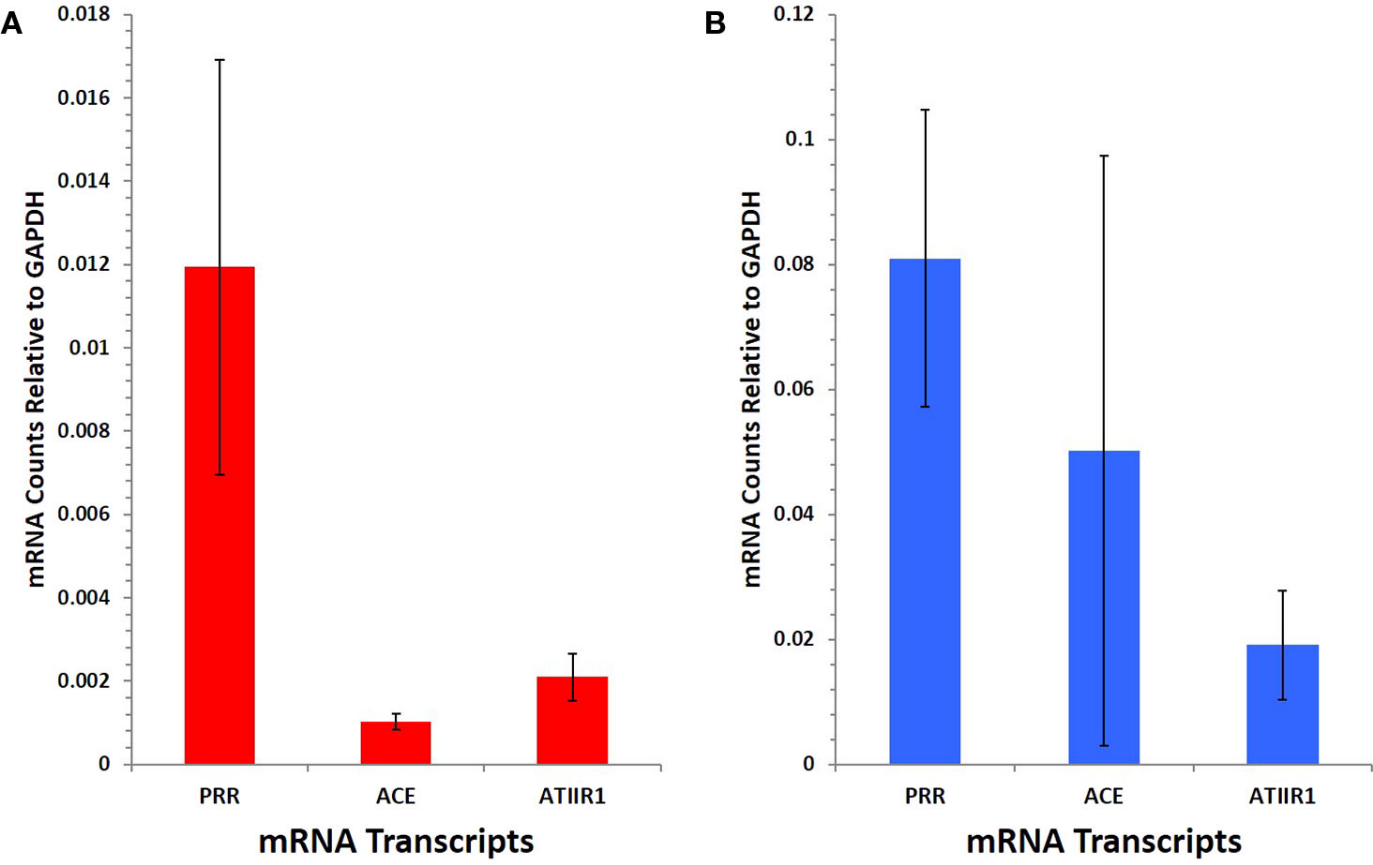
**PRR, ACE, and ATIIR1 mRNA levels in SC (*n* = 3) (A) and IM (*n* = 3) (B) VM tissues**. Total mRNA was analyzed by NanoString nCounter Gene Expression assay with specific probes for PRR, ACE, ATIIR1 and ATIIR2 genes. Counts from ATIIR2 did not exceed those of the negative control.

NanoString gene analysis showed that mRNA corresponding to PRR, ACE, and ATIIR1, but not ATIIR2, was detected in IM (Figure 5A) and SC (Figure 5B) VM tissue samples. The following average counts were calculated as expression relative to GAPDH, with their respective SEM for IM VM, PRR 0.00844 ± 0.0050, ACE 0.00064 ± 0.0002, and ATIIR1 0.00271 ± 0.0057; SC VM PRR 0.0809 ± 0.0238, ACE 0.0502 ± 0.0473, and ATIIR1 0.0191 ± 0.0087. Expression of PRR was higher in SC than IM samples, and this was statistically significant (*p* < 0.05).

## Discussion

This report provides novel findings of the presence of PRR, ACE, ATIIR1, and potentially ATIIR2 within both IM and SC VM.

The demonstration of PRR on the endothelium of VM highlights the putative role of this receptor in the biology of VM, including binding pro-renin and renin and downstream signaling through MAP kinases ERK1 and ERK2 (18). Pro-renin bound to PRR undergoes a conformational change, exposing the active sites and rendering it enzymatically active (11). Renin bound to PRR leads to a fourfold increase in catalytic activity (18). The presence of PRR on the endothelium of VM indicates a potential role for facilitating increased local conversion of angiotensinogen to ATI, indicating a role for this peptide in VM biology.

ACE has been reported as a marker for identifying primitive hemangioblasts derived from human pluripotent stem cells (21). It is exciting to speculate that the endothelium of VM, which we have demonstrated to express this marker, may reflect a relative primitive phenotype for this endothelium; however, this is the topic of our current investigation.

It is intriguing that both DAB and IF IHC analysis revealed positive staining for ATIIR2 on the endothelium as well as cells away from the endothelium, but that its expression was not demonstrated by NanoString gene expression analysis. The reasons for these include possible non-specific binding of the primary antibody used for IHC staining, despite appropriate controls being used, or that the probe used for the NanoString analysis failed to cover all the possible mRNA splice variants for this protein. Furthermore, it is interesting to note that the DAB IHC analysis of the SC and IM VM samples revealed relatively increased staining for ATIIR1 on the endothelium of smaller vessels compared to the dilated vessels, the reasons for which extend beyond this study.

The presence of PRR, ACE, ATIIR1, and potentially ATIIR2 in VM indicates a potential role for the RAS in this condition. Nguyen Dinh Cat et al. (22) have demonstrated the components of the RAS including PRR, ACE, ATIIR1, and ATIIR2 in normal vascular tissues. ATIIR1 and ATIIR2 have been shown to serve distinct functions, but share ATII as a common ligand (20). The demonstration of ATIIR1, and potentially ATIIR2, in VM presented here does not delve into the functional roles of these proteins in VM, as this is the topic of further study. However, given ATIIR1 is responsible for the proangiogenic effects of ATII (23), this may, in part, explain the increased density of abnormal venous channels within VM. The presence of ATIIR2 may indicate cellular differentiation determination, as previously proposed by Zambidis et al. (21).

A recent case report observing reduction of rectal bleeding and anemia from a VM affecting the rectosigmoid junction following administration of propranolol and celecoxib (24) was attributed by the authors to the vasoconstrictive effect of propranolol, a β-blocker, and the inhibitory effect of celecoxib, a COX-2 inhibitor, on vascular endothelial growth factor (VEGF) (24). The serendipitous observed clinical response in this case could potentially be explained by the inhibitory effects of the β-blocker and COX-2 inhibitor on the RAS. Our study demonstrates the expression of PRR on the endothelium of VM, and we hypothesize that the observed effect of these medications is *via* the reduction in plasma renin levels by a β-blocker (25) and mitigation of ATII-induced expression of PRR by a COX-2 inhibitor (26).

The putative primitive endothelial phenotype in VM is partially supported by the demonstration of positive cytoplasmic staining for stem cell growth factor receptor, *c*-kit, in small lesional vessels, but not in dilated vessels, of VM (27). We have reported a similar pattern of stronger staining for ATIIR1 on the endothelium of smaller lesional vessels, and it is exciting to speculate that the absence of *c*-kit in dilated lesional vessels may indicate that the latter are more mature and can no longer maintain a putative stem cell population, with the smaller vessels as potential precursors. However, this is a topic of our ongoing research. This observation would therefore suggest that the smaller lesional vessels are the precursors of the more mature and ectatic vessels, but this is beyond the scope of this study.

TIE2 gene mutations have been implicated in VM (7), although the exact mechanism leading to the development of VM has yet to be fully elucidated. Vikkula et al. (8) propose a role in coupling of EC proliferation and SMC recruitment.

**Figure 6:**
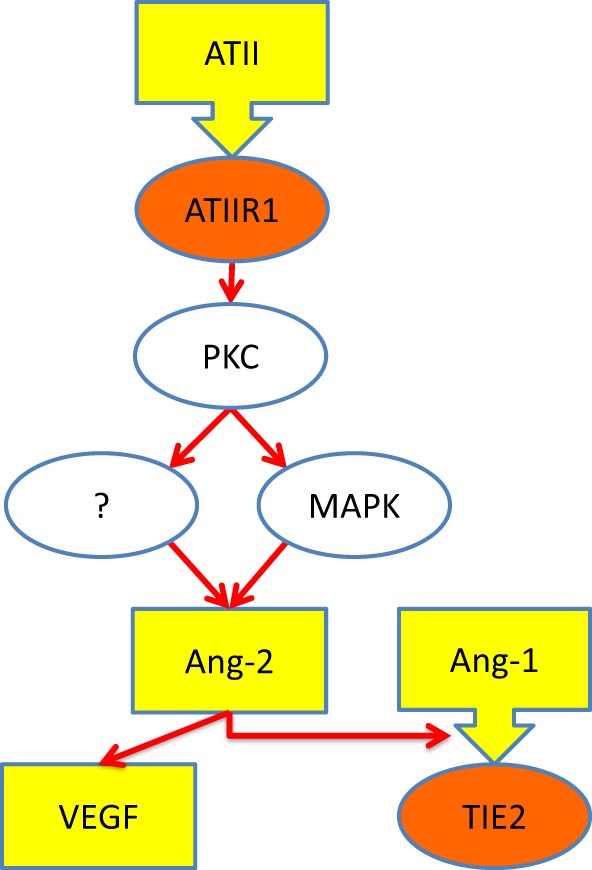
**Schematic representation of a proposed mechanism through which ATII leads to over-activation of TIE2 (see text)**. Ligands (yellow), receptors (orange), and intracellular signaling (white). Abbreviations: ATII, angiotensin II; ATIIR1, angiotensin II receptor 1; PKC, protein kinase C; MAPK, mitogen-activated protein kinase; Ang-1, angiopoetin-1; Ang-2, angiopoetin-2; VEGF, vascular endothelial growth factor.

A previous study shows that ATII, acting through ATIIR1, stimulates the production of angiopoietin-2 (Ang-2), a ligand for TIE2 (28). Given that Ang-2 modulates the effect of angiopoietin-1 (Ang-1) on TIE2 (29) as well as increases the effects of VEGF. Based on our findings, it is exciting to speculate that increased signaling *via* ATIIR1 results in increased production of Ang-2, ultimately leading to the development of VM, *via* activation of TIE2 (8, 29). Figure 6 shows our proposed model for the pathogenesis of VM incorporating a role for the RAS.

Current treatments of VM aim to relieve symptoms caused by VM, but they often neither halt nor reverse the underlying process that leads to the development and expansion of these lesions, limiting their efficacy (30), and depending on the location of the lesion, may not be feasible.

To the best of our knowledge, this is the first report demonstrating the presence of PRR, ACE, ATIIR1, and potentially ATIIR2 in IM and SC VM. This novel finding suggests the RAS as a potential therapeutic target for VM using RAS modulators.

## Declarations

The content of this article has not been submitted or published elsewhere. There was no source of funding for the article. The authors declare that there is no source of financial or other support, or any financial or professional relationships, which may pose a competing interest. All authors contributed to the preparation of this manuscript. The manuscript has been seen and approved by all authors.

## Ethics

This study was approved by the Central Health and Disability Ethics Committee (ref. no. 13/CEN/130).

## Author Contributions

TI and ST formulated the study hypothesis and designed the study. SS, EK, HDB, TI and STT analyzed IHC data. SS and TI analyzed the NanoString data. RM performed statistical analysis. SS, EK, TI and STT drafted the manuscript. All authors read and approved the manuscript.

## Conflict of Interest Statement

The authors declare that the research was conducted in the absence of any commercial or financial relationships that could be construed as a potential conflict of interest.

## Supplementary Material

Figure S1 **Representative DAB IHC-stained sections of IM (A) and SC (B) VM stained for D2-40 (brown) demonstrating no immunoreactivity except for normal lymphatic vessels in the periphery**. Original magnification: 100×.

Figure S2 **Positive controls for DAB IHC staining: placenta for PRR [(A), brown], kidney for ACE [(B), brown], liver for ATIIR1 [(C), brown], and kidney for ATIIR2 [(D), brown] and a section of VM as a negative control by omitting the primary antibody [(E), brown]**. Original magnification: 400×.

Figure S3 **Images of IF IHC-stained sections of IM (A,C,E,G,I,K,M,O) and SC (B,D,F,H,J,L,N,P) VM, showing expression of PRR [(A,B), red], ACE [(E,F), green], ATIIR1 [(I,J), green], and ATIIR2 [(M,N), red]**. Endothelial cells were marked by either CD34 [(C,D,O,P), green] or ERG [(G,H,K,L), red] staining. Cell nuclei were counterstained with 4′,6-diamidino-2-phenylindole (blue). Scale bars: 20 μm.

Figure S4 **Negative controls for IF IHC staining, demonstrating appropriate specificity of primary antibodies in anti-mouse [(A), green] and anti-rabbit [(B), red] combinations**. Scale bars: 20 μm.
