# Supplementary figures and images for: Expression of the Components of the Renin–Angiotensin System in Venous Malformation
Source: Front Surg. 2016 May 3;3:24. doi: 10.3389/fsurg.2016.00024 (PMC4853390; doi:10.3389/fsurg.2016.00024)

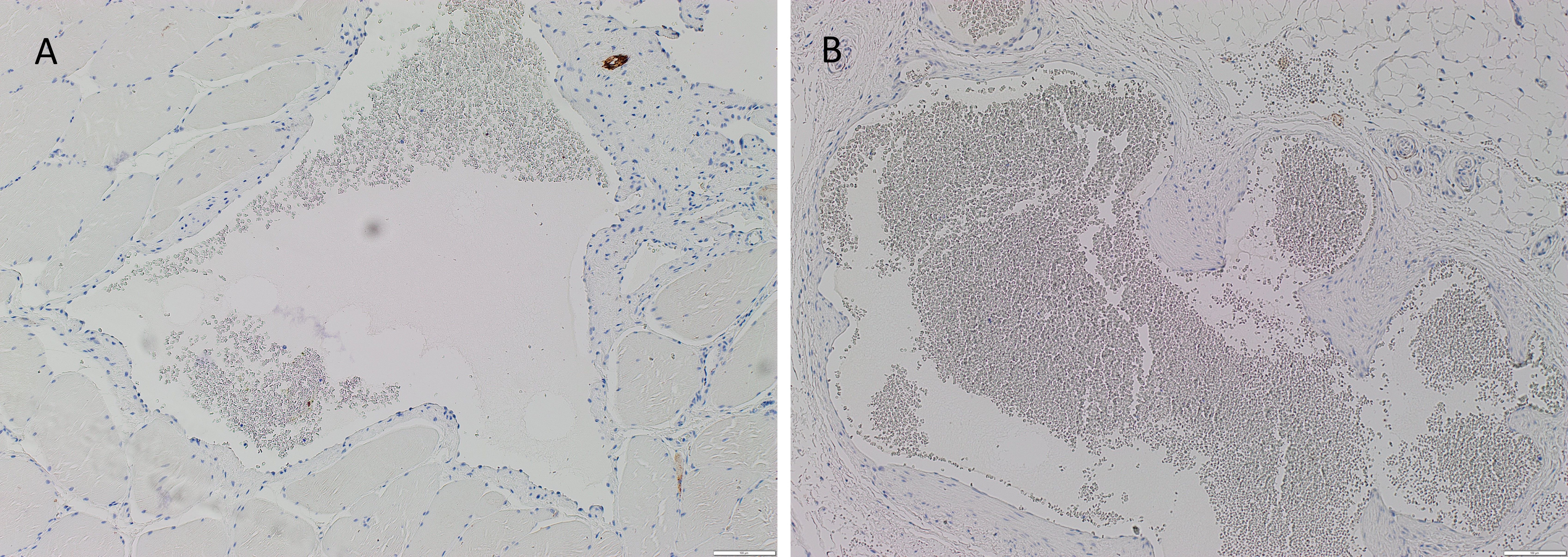

Supplement: Figure S1 — Representative DAB IHC-stained sections of IM (A) and SC (B) VM stained for D2-40 (brown) demonstrating no immunoreactivity except for normal lymphatic vessels in the periphery. Original magnification: 100×. [file image_1.jpg]

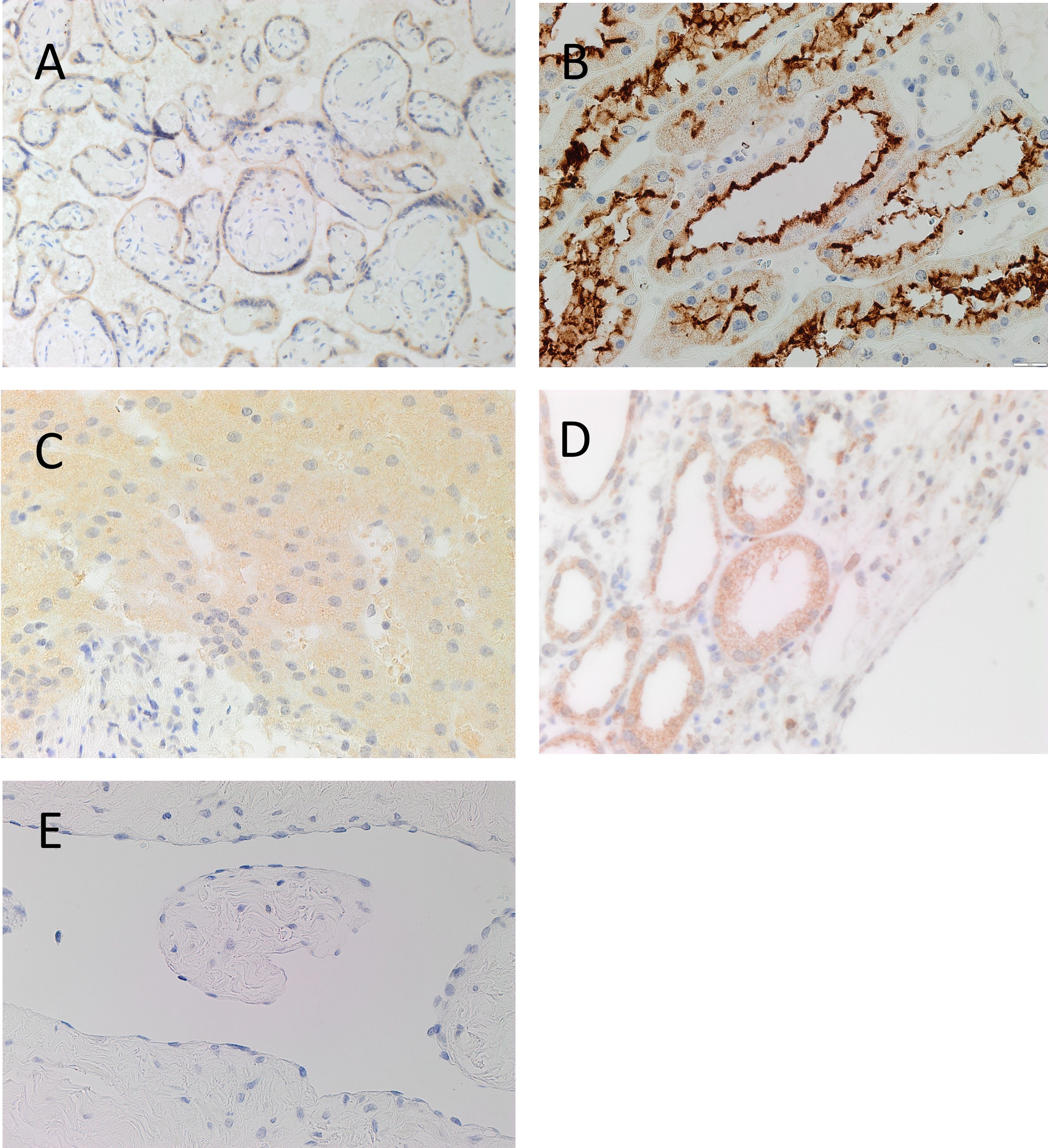

Supplement: Figure S2 — Positive controls for DAB IHC staining: placenta for PRR [(A), brown], kidney for ACE [(B), brown], liver for ATIIR1 [(C), brown], and kidney for ATIIR2 [(D), brown] and a section of VM as a negative control by omitting the primary antibody [(E), brown]. Original magnification: 400×. [file image_2.jpg]

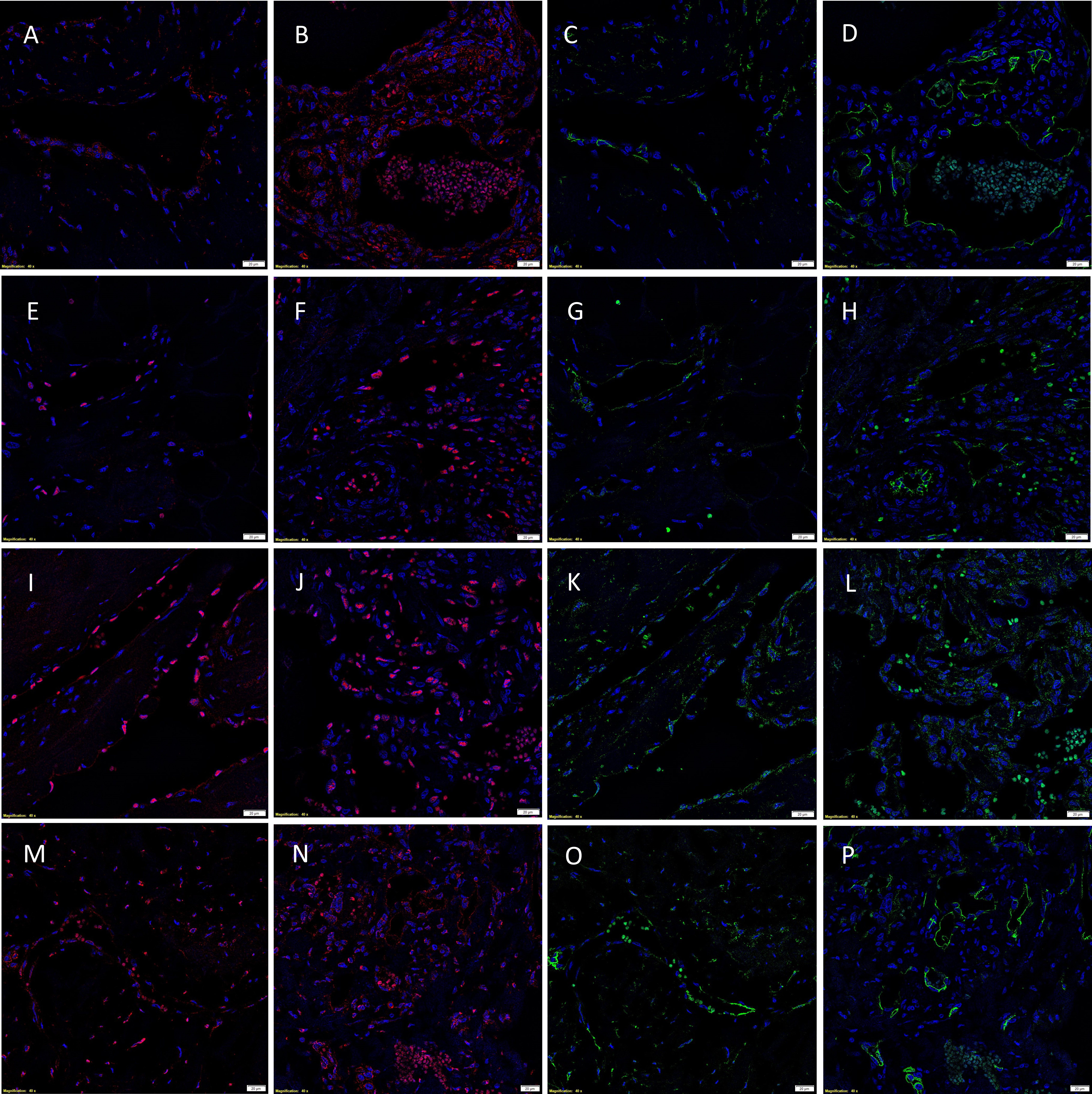

Supplement: Figure S3 — Images of IF IHC-stained sections of IM (A,C,E,G,I,K,M,O) and SC (B,D,F,H,J,L,N,P) VM, showing expression of PRR [(A,B), red], ACE [(E,F), green], ATIIR1 [(I,J), green], and ATIIR2 [(M,N), red]. Endothelial cells were marked by either CD34 [(C,D,O,P), green] or ERG [(G,H,K,L), red] staining. Cell nuclei were counterstained with 4′,6-diamidino-2-phenylindole (blue). Scale bars: 20 μm. [file image_3.jpg]

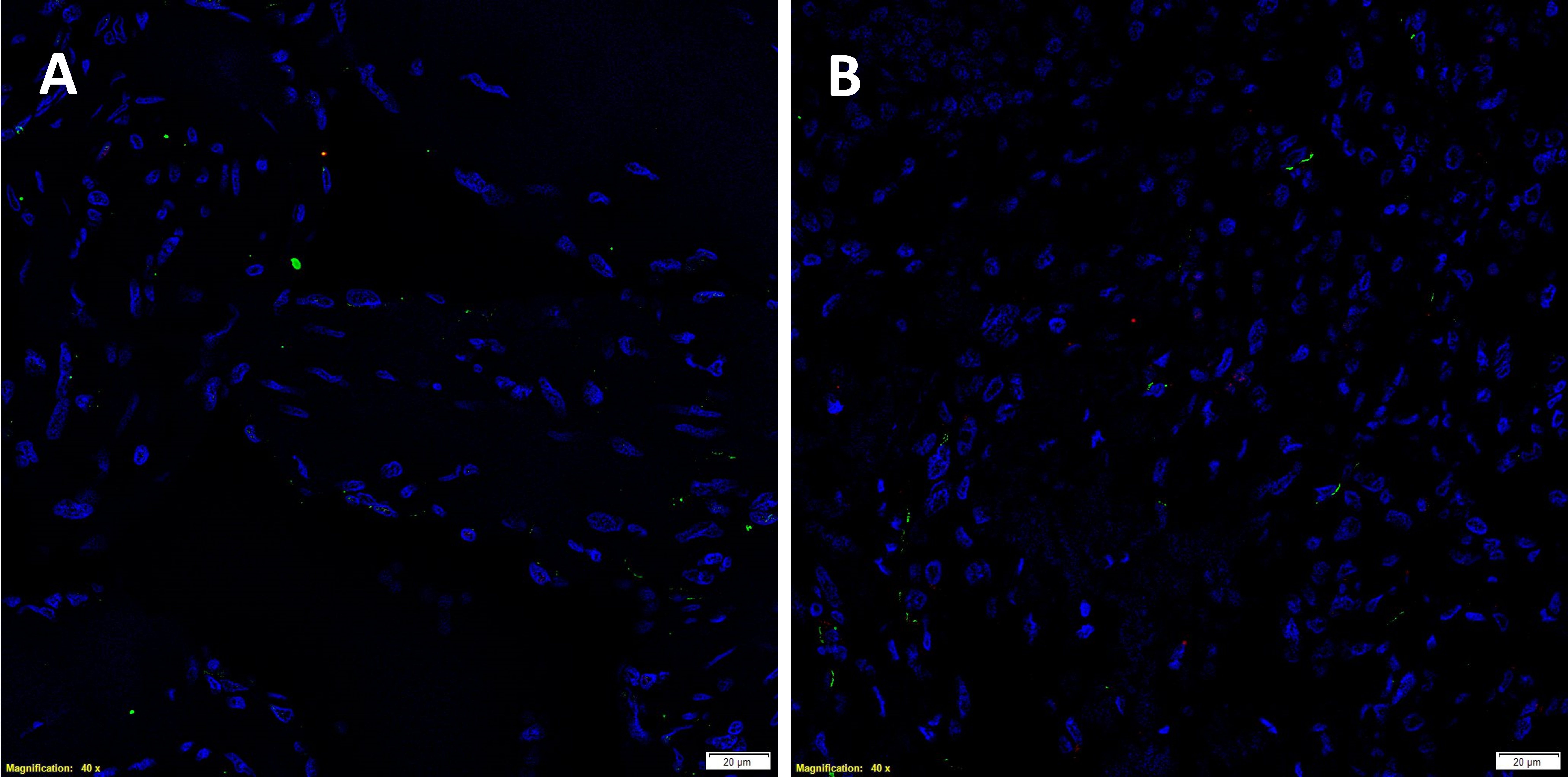

Supplement: Figure S4 — Negative controls for IF IHC staining, demonstrating appropriate specificity of primary antibodies in anti-mouse [(A), green] and anti-rabbit [(B), red] combinations. Scale bars: 20 μm. [file image_4.jpg]
